# ENaC blockade in proteinuria‐associated extracellular fluid volume overload – effective but risky

**DOI:** 10.14814/phy2.13835

**Published:** 2018-09-03

**Authors:** Evan C. Ray

**Affiliations:** ^1^ Renal‐Electrolyte Division Department of Medicine School of Medicine University of Pittsburgh Pittsburgh Pennsylvania

Renal Na retention and edema represent cardinal features of proteinuric kidney disease. Clinical data suggest that, in some patients with heavy proteinuria, nephrotic syndrome, renal Na retention, and extracellular fluid volume overload occur in spite of a suppressed renin‐aldosterone system (Meltzer et al. 1979). Although systemic factors may contribute to urinary Na retention, a rat unilateral kidney model of nephrotic syndrome demonstrated that mechanisms intrinsic to the diseased kidney contribute to Na retention (Ichikawa et al. 1983). Proteinuria stimulates Na re‐absorption in the aldosterone‐sensitive distal nephron. This Na‐reabsorption is sensitive to blockers of the epithelial sodium channel (ENaC), suggesting ENaC plays a role in proteinuria‐associated Na‐retention (Deschenes et al. 2001).

The last decade has led to significant advances in our understanding of the regulation of ENaC and insights regarding mechanisms by which proteinuria may enhance ENaC activity. Ten years ago, researchers at the University of Pittsburgh and at the University of Southern Denmark independently described the ability of the serine protease, plasmin, to activate ENaC (Passero et al. 2008; Svenningsen et al. 2009). Plasmin's precursor, plasminogen, can be leaked from the bloodstream into the tubular ultrafiltrate through damaged glomeruli and may be activated by urokinase expressed in the tubule. In channels where the *γ* subunit has already undergone cleavage by the intracellular protease, furin, secondary cleavage by plasmin allows removal of an inhibitory tract between the cleavage sites. Loss of the inhibitory tract increases the channel's open probability (Ray et al. 2015). A host of other secondary proteases, if present, could also perform this role and activate ENaC. However, for extracellular proteases to access the channel, ENaC must be present at the cell surface. Mechanisms that maintain ENaC expression and cell surface delivery in the context of proteinuria‐associated extracellular fluid volume overload remain unclear. Interestingly, subjects with membranous nephropathy are unresponsive to mineralocorticoid antagonism (Usberti and Gazzotti 1998). This observation suggests that activation of ENaC in these patients may be independent of canonical mineralocorticoid signaling. Mechanistically, this distinguishes at least some types of nephrotic syndrome from other disorders characterized by total body Na overload, such as cirrhosis and heart failure. In these disorders, aldosterone signaling plays a key pathophysiologic role, and mineralocorticoid receptor antagonism represents an important therapeutic modality.

In this issue of Physiological Reports, Gitte Hinrichs and colleagues at the University of Southern Denmark re‐visit activation of ENaC in proteinuria. They describe their experience using the ENaC antagonist, amiloride, in a patient with diabetes and nephrotic‐range proteinuria (Hinricks et al. 2018). Although amiloride is generally considered a rather poor anti‐hypertensive agent, prescription of 5 mg of amiloride per day to their patient was associated with significant diuresis and a 25 mmHg reduction in systolic blood pressure. The authors point out that others have reported brisk diuresis in patients with proteinuria in response to ENaC blockade, but their observations further support an important role for ENaC in proteinuria‐associated Na retention.

Hinricks et al. (2018) further observed that this patient's hypertension and edema failed to respond to spironolactone. Although patient adherence to this medication prescription was not proven, this finding is consistent with mineralocorticoid‐independent enhancement of renal Na retention, similar to previous observations in membranous nephropathy.

Unfortunately, the patient also experienced acute kidney injury and dramatic hyperkalemia. Because he was concurrently prescribed an angiotensin‐converting enzyme inhibitor, a mineralocorticoid antagonist, and a loop diuretic, these adverse effects may not be solely attributable to amiloride. Nonetheless, the findings are consistent with observations from a randomized cross‐over controlled trial that compared amiloride and hydrochlorothiazide in subjects with type 2 diabetes and proteinuria (Unruh et al. 2017). This study was terminated early when amiloride induced acute kidney injury and hyperkalemia in two of the first nine subjects who were recruited. The rapid diuresis experienced by Hinrichs’ patient suggests that reduced renal perfusion may have contributed to the acute kidney injury. In the study by Unruh and colleagues, decline in extracellular fluid volume was less clear. Another intriguing possibility is that loss of ENaC activity may have hemodynamic consequences. Amiloride‐sensitive Na entry in the connecting tubule appears to promote vasodilation of the afferent arteriole (Wang et al. 2010). ENaC blockade may promote afferent arteriolar vasoconstriction, with reduced glomerular perfusion and filtration. Ultimately, Hinrichs and colleagues were able to maintain their patient on a regimen of low‐dose amiloride (2.5 mg/day), in combination with an angiotensin converting enzyme inhibitor, a thiazide diuretic, and a loop diuretic.

This report supports the theory that proteinuria stimulates ENaC in a mineralocorticoid‐receptor independent fashion. Furthermore, these findings suggest that blockers of ENaC, such as amiloride, may be effective tools in treating hypertension and edema in nephrotic syndrome. However, the apparent potential for acute kidney injury and hyperkalemia demand great caution with this approach, lest we risk use of a remedy that is worse than the disease.

## Funding Information

Funding provided by the National Institutes of Health: K08 DK110332.
